# The prognostic value of tumor mutational burden and immune cell infiltration in esophageal cancer patients with or without radiotherapy

**DOI:** 10.18632/aging.102917

**Published:** 2020-03-12

**Authors:** Cheng Yuan, Liyang Xiang, Kuo Cao, Jianguo Zhang, Yuan Luo, Wenjie Sun, Nannan Zhang, Jiangbo Ren, Junhong Zhang, Yan Gong, Conghua Xie

**Affiliations:** 1 Department of Radiation and Medical Oncology, Zhongnan Hospital of Wuhan University, Wuhan 430071, Hubei, China; 2 Department of Biological Repositories, Zhongnan Hospital of Wuhan University, Wuhan 430071, Hubei, China; 3 Hubei Key Laboratory of Tumour Biological Behaviors, Zhongnan Hospital of Wuhan University, Wuhan 430071, Hubei, China; 4 Hubei Cancer Clinical Study Center, Zhongnan Hospital of Wuhan University, Wuhan 430071, Hubei, China; 5 Human Genetics Resource Preservation Center of Hubei Province, Human Genetics Resource Preservation Center of Wuhan University, Zhongnan Hospital of Wuhan University, Wuhan 430071, Hubei, China

**Keywords:** esophageal cancer, tumor mutational burden, radiotherapy, immune cell infiltration

## Abstract

Growing evidence highlighted the tumor mutational burden (TMB) as an important feature of carcinogenesis and therapeutic efficacy in esophageal cancer (EC). Our study aimed to explore the genomic landscape and the correlation between TMB and immune cell infiltration in EC patients with or without radiotherapy. The EC patients were categorized into high TMB (TMB-H) and low TMB (TMB-L) groups by the ESTIMATE algorithm, and subgroup analysis was performed based on receiving radiotherapy or not. Univariate regression analysis indicated TMB and TNM stages as high-risk prognostic factors (Hazard ratio > 1 and P < 0.05). Multivariate regression analysis suggested TMB as an independent prognostic factor (Hazard ratio = 1.051, P = 0.003). Kaplan-Meier analysis showed no significant difference of the overall survival (OS) between TMB-H and TMB-L groups (P = 0.082). However, EC patients without radiotherapy in the TMB-H group had significantly decreased OS (P = 0.038) and increased Tregs cell infiltration (P = 0.033). These results suggested TMB as a prognostic marker for EC patients. Especially for patients who did not receive radiotherapy, the prognosis of TMB-H patients was significantly poorer than that of TMB-L patients, which might result from the different regulatory T cell infiltration.

## INTRODUCTION

Esophageal cancer (EC) is one of the most prevalent malignancies and common causes of cancer-related death globally [1]. The main pathological subtypes included esophageal adenocarcinoma (EA) and esophageal squamous cell carcinoma (ESCC). ESCC accounts for 90% of EC in Asian countries, including China and Japan [2]. Although great advances in treatment were achieved in last decades, the prognosis of EC is still unsatisfactory [3, 4].

Cancer is the phonotypic end point of accumulated genetic and epigenomic alterations [5]. Many endogenous and exogenous factors, such as DNA damage repair inactivation, DNA erroneous replication, microsatellite instability, and carcinogen exposure, lead to increased somatic mutations [6]. The total number of mutations occurring in a tumor specimen is termed tumor mutation burden (TMB), which sketches out the status of genomic mutation [6]. Increasing attentions have been drawn to the linkage of genomic mutation profiling to patient characteristics with clinical outcome recently.

Previous evidence suggested that higher TMB was likely to harbor more neoantigens as targets for activated immune cells [7]. The impacts of the TMB on tumor progression or immune infiltration are still to be investigated [8]. Many studies revealed the link between immunotherapy responses and TMB [9–12]. While not all mutations generate immunogenic, only a few mutations can be recognized by T cells [13–15]. Therefore, understanding the immune cell composition and function is critical to effectively manage cancer progression and immune response.

In the presented study, we analyzed the difference of clinical features, such as ages, genders, tumor grades, tumor stages, races and radiation, between high TMB (TMB-H) and low TMB (TMB-L) groups. Then, we evaluated the genomic landscape of EC patients, and their associations with clinical parameters (genders, TNM stages, T stage, N stage and M stage) and overall survival (OS). Additionally, the correlation between TMB and immune cell infiltration was analyzed in EC patients with or without radiotherapy. Our analysis showed that TMB might be a potential prognostic assessment marker. Especially for patients not receiving radiotherapy, the prognosis of TMB-H patients was significantly poorer than that of TMB-L patients, which might result from the different degrees of regulatory T cell infiltration.

## RESULTS

### Mutational genomic landscape

**Figure 1:**
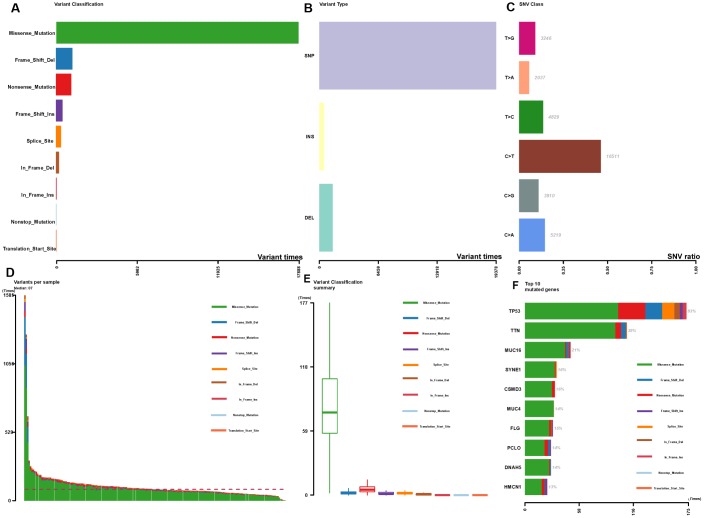
**Summary of the mutation information with statistical calculations.** (**A**–**C**) Classification of mutation types according to different categories, in which missense mutation accounts for the most fraction, SNP showed more frequency than insertion or deletion, and C>T was the most common of SNV; (**D**, **E**) TMB in specific samples; (**F**) the top 10 mutated genes in EC.

**Figure 2:**
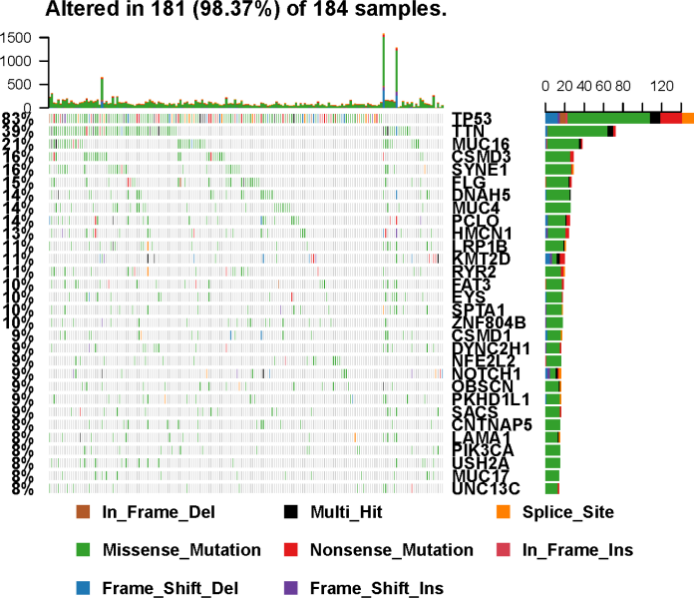
**Landscape of mutation profiles in EC samples.** Mutation information of each gene in each sample was shown in the waterfall plot, in which various colors with annotations at the bottom represented the different mutation types. The barplot above the legend exhibited the mutational burden.

The summary of mutational genomic landscape was shown in Figure 1. By calculating the 9 variant classifications separately, missense mutation had the highest mutation frequency in the total mutation frequency (Figure 1A). In addition, single nucleotide polymorphism (SNP) was more common than INS and DEL (Figure 1B). Single nucleotide variants (SNVs) were classified into 6 base substitutions (C > A, C > G, C > T, T > A, T > C and T > G), and the results indicated C > T had the highest incidence (16511 times, Figure 1C). The values of variants varied from 0 to 1585, with a median value of 87 (Figure 1D). Missense mutation had the highest mutation frequency in personal mutation frequency (Figure 1E). We calculated the genes with the highest mutational rate, and the top 10 mutated genes were as follows: TP53, TTN, MUC16, SYNE1, CSMD3, MUC4, FLG, PCLO, DNAH5 and HMCN1 (Figure 1F). The waterfall map summarized the high mutated genes and their mutation classification (Figure 2). In addition, the proportion of more mutated genes was visualized using the genecloud (Supplementary Figure 1). Genes with mutation frequency ≥ 5 times were presented in the genecloud, which was consistent with the results in Figure 1F and Figure 2. These high mutated genes might be functionally related, therefore, we further studied their interaction. The co-occurrence and exclusive relationships between these mutant genes were shown in Supplementary Figure 2. The co-occurrence correlation between RYR2 and FLG was the most significant (P < 0.001).

### An overview of the clinical implications associated with TMB

These 182 patients included in this study were consisted of 95 ESCC and 87 EA. The general characteristics of patients with EC were shown in Table 1. To determine the critical value of TMB, population was divided into 2 groups by mean values. The clinical factors, such as ages, genders, tumor grades, tumor stages, races and radiation, were not associated with TMB levels (P > 0.05). In addition, the level of TMB in EA was significantly higher than that in ESCC, but there was no significant difference of OS between the 2 tumor subtypes (Supplementary Figure 3).

**Table 1:** General characteristics of patients with esophageal cancer.

**Characteristics**	**TMB-L group (n = 134)**	**TMB-H group (n = 48)**	**ES**	**P value**
Age (years)	62.95 (28.01-90.06)	68.61 (44.02-90.06)	-2.265	0.605
Gender			0.179	0.677
Male (%)	115 (85.82%)	40 (83.33%)		
Tumor grade (n)			4.431	0.219
G1	13	6		
G2	60	15		
G3	36	12		
unknow	25	15		
Stage (n)			1.945	0.746
I	15	3		
II	57	21		
III	37	17		
IV	7	2		
unknow	18	5		
Race			7.712	0.052
White	79	33		
Asian	39	7		
Black or African American	5	0		
Unknown	11	8		
Radiation			1.288	0.525
Yes	15	3		
No	96	38		
unknow	23	7		

TMB: tumor mutation burden; TMB-H: high TMB; TMB-L: low TMB; ES: effect size.

**Figure 3:**
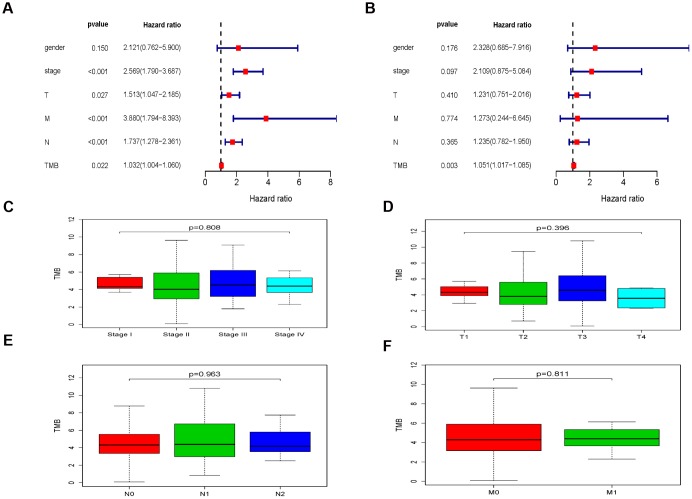
**The clinical implications associated with TMB.** (**A**) The univariate regression analysis of TMB and clinical information. (**B**) The multivariate regression analysis of TMB and clinical information. (**C**–**F**) The relationship between TMB and TNM stages.

After integrating TMB and clinical information (genders, TNM stages, T stage, N stage and M stage), univariate and multivariate regression analysis on the impacts of prognosis were performed. The univariate regression analysis indicated that TMB, TNM stages, T stage, N stage and M stage were high-risk prognostic factors (hazard ratio > 1 and *P* < 0.05, Figure 3A). Multivariate regression analysis suggested TMB as the independent prognostic factor (hazard ratio = 1.051, P = 0.003, Figure 3B). Then, we further studied the relationship between TMB and TNM stages, and found that there was no significant correlation between TMB and TNM stages (TNM stage: P = 0.808, Figure 3C; T stage: P = 0.396, Figure 3D; N stage: P = 0.963, Figure 3E and M stage: P = 0.811, Figure 3F).

**Figure 4:**
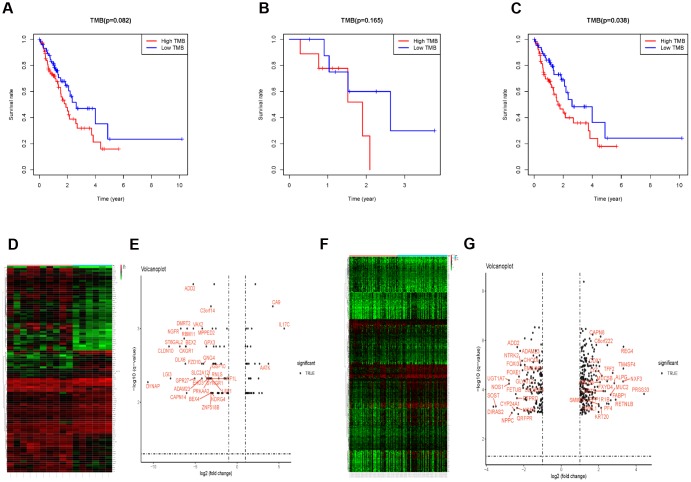
**The subgroup analysis in patients receiving radiotherapy or not.** (**A**) Survival analysis to explore the OS of EC patients between the TMB-H and TMB-L group. (**B**) OS between the TMB-H and TMB-L group of patients receiving radiotherapy. (**C**) OS of the TMB-H group decreased significantly in EC patients with radiotherapy. (**D**–**G**) The different gene expression between the TMB-H and TMB-L groups in EC patients receiving radiotherapy or not.

Kaplan-Meier analysis indicated that there was no significant difference of OS between the TMB-H group and TMB-L groups (P = 0.082, Figure 4A). Considering the effects of radiotherapy on TMB and prognosis, we performed a subgroup analysis based on whether they received radiotherapy or not. There was no significant difference of OS between the TMB-H and TMB-L group in EC patients receiving radiotherapy (P = 0.165, Figure 4B), however, OS of the TMB-H group decreased significantly in EC patients who did not receive radiotherapy (P = 0.038, Figure 4C).

### Immune infiltration between the TMB-H group and TMB-L group

**Figure 5:**
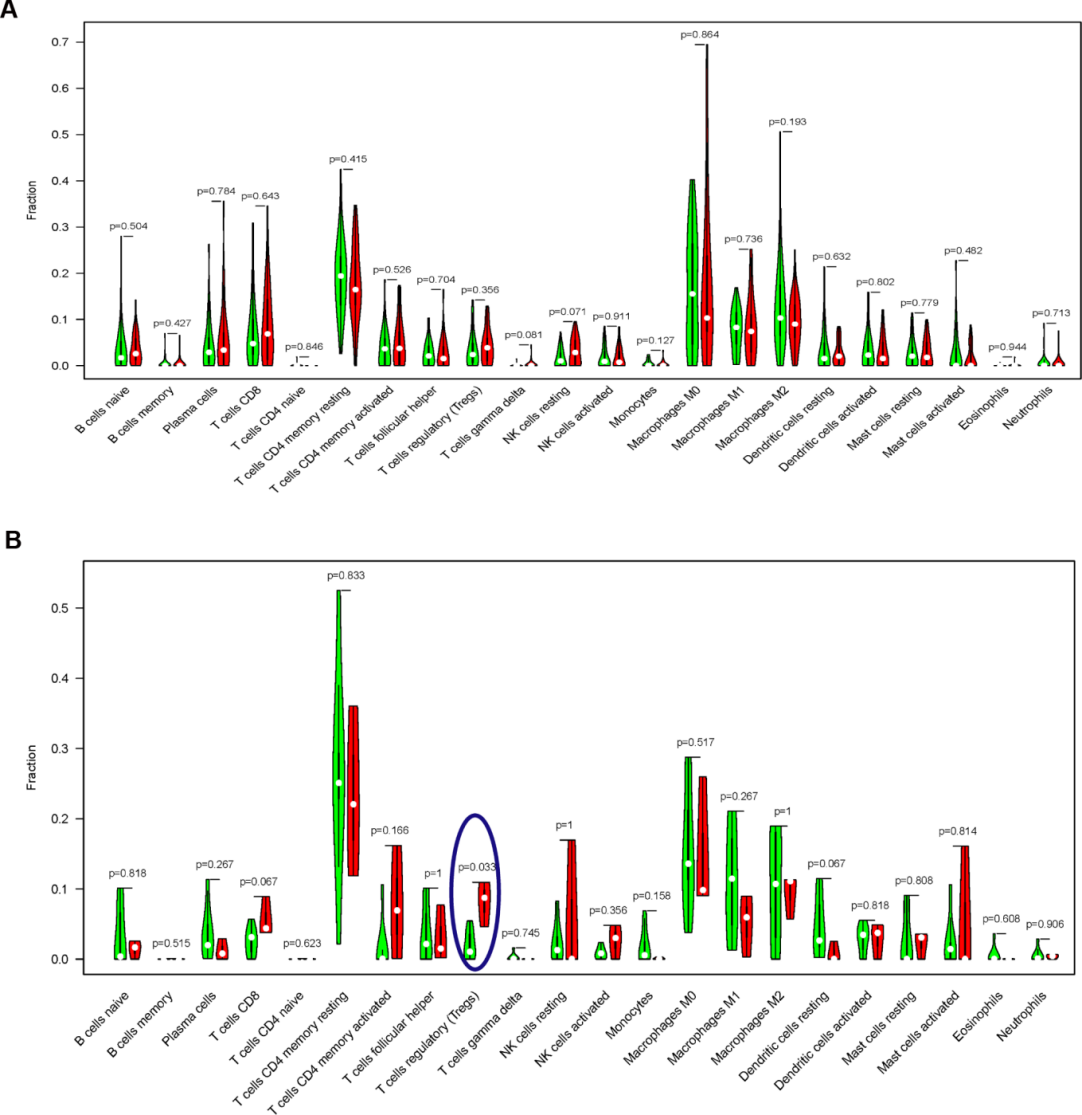
**Comparisons of 22 important immune fractions between the TMB-H and TMB-L groups.** (**A**) No significant difference of the 22 important immune cell infiltration between the TMB-H and TMB-L groups in EC patients with radiotherapy; (**B**) The levels of Tregs cell infiltration in the TMB-L group were lower compared with those in the TMB-H group of EC patients without radiotherapy.

Prior to the immune infiltration, differences of gene expression between the TMB-H and TMB-L groups were calculated and visualized by “limma” package (Radiotherapy: Figure 4D and 4E; without Radiotherapy: Figure 4F and 4G). Based on this differentially expressed data, we used the deconvolution method to calculate the difference of immune cell infiltration between the TMB-H and TMB-L groups. The TMB-H group had significantly increased Tregs cell infiltration in EC patients who did not receive radiotherapy (P = 0.033), while there was no significantly different infiltration of 22 kinds of immune cells among patients receiving radiotherapy (Figure 5).

## DISCUSSION

With the rapid development of microarray sequencing, researchers are increasingly exploring new targets and performing external validations using statistical algorithms in cancer. However, most current studies have not effectively classified and analyzed the components of immune cells and the TMB, which may markedly affect the characteristics of cancer treatment response, especially precision radiotherapy. The CheckMate 227 trial recently established a TMB of ≥10 mutations per 10^6^ bases as a robust and independent biomarker of response [16]. These data recently led radiation oncologists to speculate whether the improved efficacy was caused by radiation-induced TMB. As a tumor with high mutational loads, EC was estimated to have 3,000 to 300,000 mutations per tumor [17, 18]. Therefore, we attempted to explore immune cell components and degrees of TMB, extracting significant biomarkers of large prognostic value to understand tumor progression in EC patients with or without radiotherapy.

We constructed a model based on the TMB status and classified EC patients into TMB-H and TMB-L groups by the mean of population TMB. Patients with less TMB had a better prognosis, suggesting that TMB as a risk-independent prognostic factor. Cigarette smoking was identified as an important pathogenic factor for ESCC [19]. The TMB-H group was composed of smoking, aging and other cancer risk factors related patients, which accorded with the results of adverse prognosis. Our study suggested that TMB was an important prognostic factor for EC, however, there was no difference of survival analysis results. To explain this contradiction, subgroup analysis was performed based on whether patients received radiotherapy or not.

Our study is the first to analyze the correlation between radiotherapy and TMB in EC patients. For EC patients who did not receive radiotherapy, the OS of the TMB-L group was significantly prolonged. Excluding the interference of radiotherapy, TMB was a prognostic factor. Radiotherapy might improve the impact of TMB on EC via regulating immune infiltration. Treg cell infiltration was induced in the TMB-H group of EC patients who did not receive radiotherapy. Treg cell plays a central role for maintenance of immune homeostasis and self-tolerance [20–23]. It was well recognized that the immunosuppression caused by the accumulation of Treg cells in the tumor microenvironment led to poor prognosis in cancer patients [24–27]. In addition, Treg cells were also important prognostic variables in patients receiving radiotherapy [28]. Previous studies reported that the numbers of Treg cells and their immunosuppressive functions were both increased after radiotherapy [29–32]. Treg cells were reported to weaken the clearance of cancer cells by radiotherapy and induce resistance to radiotherapy [33].

TMB is associated with the abundance of neoantigens and increased immunogenicity [34, 35], as well as increased immunogenicity [36]. High TMB reflects the presence of mutation-associated neoantigens, with consequent increased lymphocyte infiltration in the tumor microenvironment [37, 38]. Treg cell infiltration in the TMB-H group was significantly higher than that in the TMB-L group for EC patients without radiotherapy, while there was no difference of Treg cell infiltration in EC patients receiving radiotherapy. Whether radiotherapy balanced the Treg cell infiltration, or Treg cell infiltration was affected by other characteristics of EC patients receiving radiotherapy still needs to be further explored.

It should be noted that the correlation between the infiltration of immune cells and the level of TMB was not consistent in different tumors. For example, most of the immune signatures were upregulated in the TMB-L subtype, while downregulated in the TMB-L subtype of cervical squamous cell carcinoma. The Treg cells were inclined to be upregulated in the TMB-L subtype of various cancer types, however, we found that high TMB was associated with elevated Treg cell infiltration in EC patients without radiotherapy. Some studies suggested that high TMB resulted in numerous neoantigens that incited anti-tumor immune responses [11], and that high TMB was associated with genomic instability, resulting in induced anti-tumor immune responses [39]. Another reason behind the discrepancy could be that most of the TCGA patients were not treated with immunotherapy. Indeed, for the TCGA cases likely with immunotherapy such as melanoma, higher TMB was associated with better prognosis.

There were some limitations in our research that could not be ignored. First, the number of patients receiving radiotherapy reported in the TCGA database was small. The limited sample size led to instability of statistical results. Second, we did not exclude the extreme value of poor prognosis (such as OS < 90 days). The existence of these data would most likely interfere with the outcome of survival analysis.

Together, we performed a comprehensive analysis on TMB in EC, and our results suggested that TMB could be considered as a prognostic marker in the patients who did not receive radiotherapy. The prognosis of the TMB-H patients was significantly lower than that of TMB-L, which might be related to the difference in Treg cell infiltration. Further studies are needed to characterize molecular subtyping based on TMB and to explore potential relationships between Treg cell infiltration and TMB.

## MATERIALS AND METHODS

### Data extraction from dataset

All the EC patients’ somatic mutations data, transcriptome sequencing data, and clinical information were downloaded and collected from The Cancer Genome Atlas (TCGA, https://portal.gdc.cancer.gov/). TMB was defined by the number of somatic mutations per genomic area for target sequencing. Specifically, TMB is calculated by the total number of somatic mutations / total covered bases.

### Mutation signature analysis

Mutation signature analysis was performed to devolve cancer somatic mutation counts, stratified by mutation contexts or biologically meaningful subgroups, into a set of characteristic signatures and to infer the pattern of each discovered signatures across samples. All SNVs were classified into 96 possible mutation categories based on the 6 base substitutions (C > A, C > G, C > T, T > A, T > C and T > G) and 16 possible combinations of neighboring bases within the trinucleotide sequence context. In order to compare the eigenvalues of the TBM-L group and TMB-H group, Wilcoxon test was used to identify the difference of mutation characteristics.

### Gene expression analysis

The raw biological data of DNA microarray were preprocessed and normalized to remove bias and ensure the uniformity and integrity. Background correction, propensity analysis, normalization and visualization output of probe data were then performed with robust multi-array average analysis algorithm in “limma” package. Differential expression genes (DEGs) were determined between the TBM-L and TMB-H group. The cut-offs were (|log2(FC)| > 1 and P value < 0.05.

### Analysis of the correlation between TMB and clinical data

Clinicopathological parameters including TNM stages in EC patients were analyzed and displayed according the TMB scores. The Wilcox test (gender, M stage and TMB) or Kruskal test (TNM stages, T stage and N stage) was utilized to measure statistically significance. The primary end point of survival comparison for EC patients was OS, which was evaluated from the date of first therapy to the date of death or last follow-up. The EC patients were divided into 2 subgroups based on whether they received radiotherapy or not. The prognosis of the 2 subgroups was then analyzed separately. The follow-up duration was estimated using the Kaplan-Meier method and log-rank test in distinct curves. All hypothetical tests were two-sided and P value < 0.05 was considered significant in all tests.

### Immune infiltration

The relative levels of distinct immune cell types were quantified using CIBERSORT within a complex gene expression mixture. Each immune cell subtype was characterized and quantified using gene expression signatures consistent of ~500 genes in CIBERSORT. Here, gene expression datasets were prepared using standard annotation files and data uploaded to the CIBERSORT web portal (http://cibersort.stanford.edu/), with the algorithm run using the default signature matrix at 1,000 permutations.

## Supplementary Material

Supplementary Figures
