# Supplementary material for: The prognostic value of tumor mutational burden and immune cell infiltration in esophageal cancer patients with or without radiotherapy
Source: Aging (Albany NY). 2020 Mar 12;12(5):4603–16. doi: 10.18632/aging.102917 (PMC7093160; doi:10.18632/aging.102917)
Supplement: Supplementary Figures [file aging-12-102917-s001..pdf]

SUPPLEMENTARY FIGURES

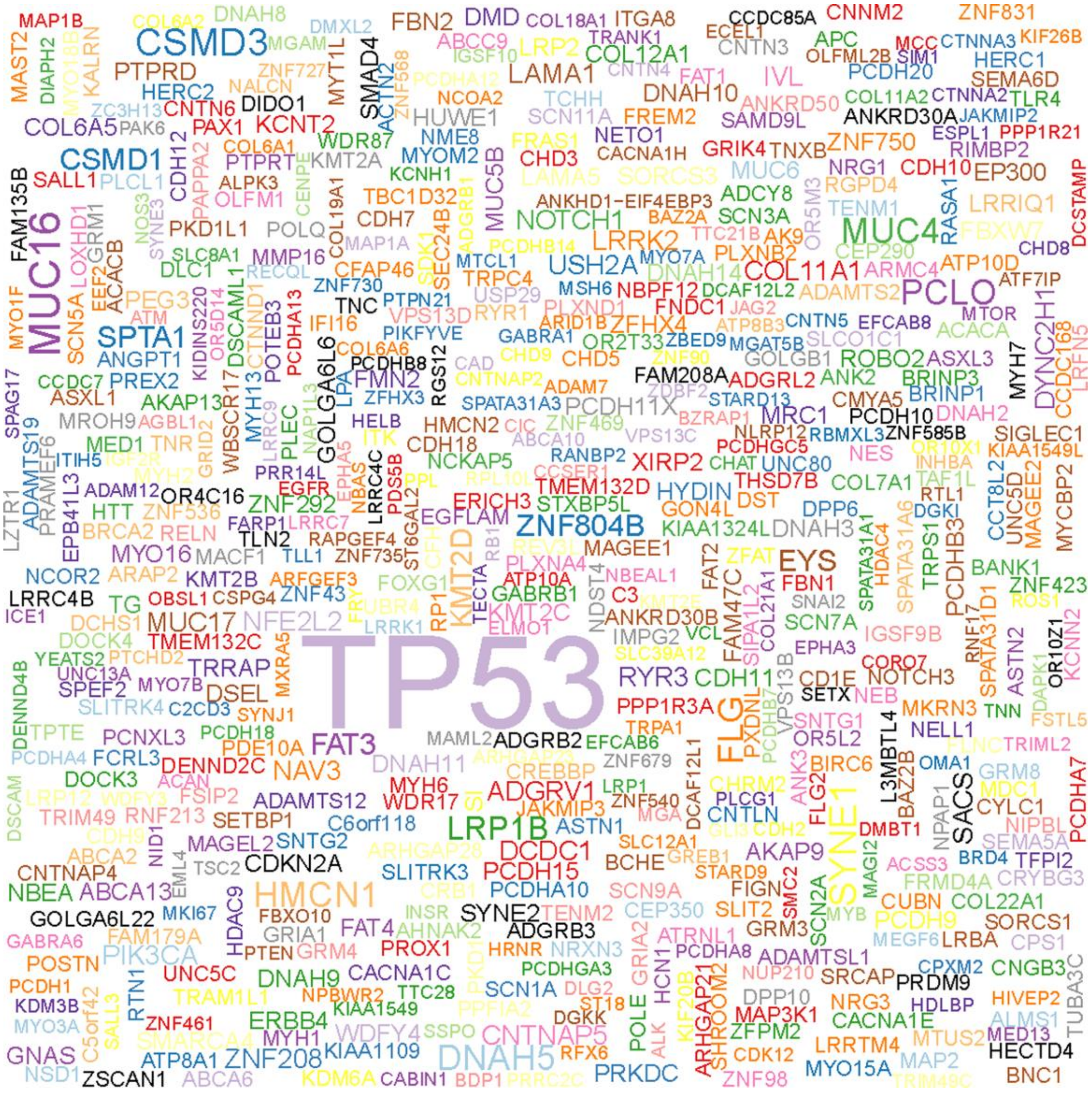

Supplementary Figure 1. Gene cloudmap. The size of the gene symbol represents the frequency of mutations.

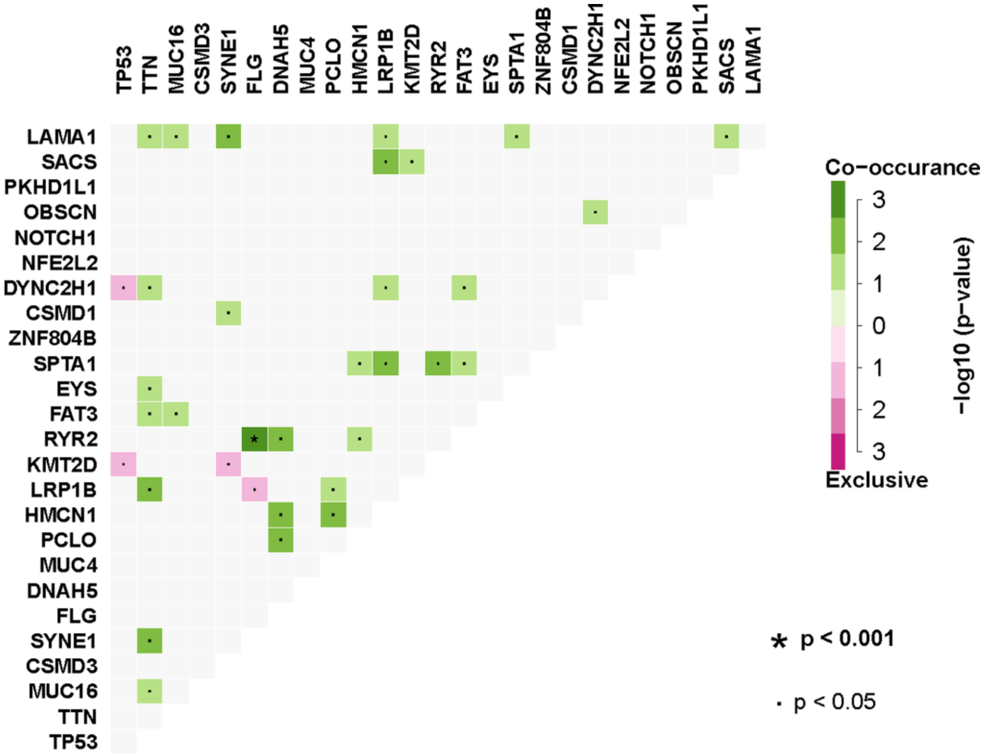

Supplementary Figure 2. The coincident and exclusive associations across mutated genes.

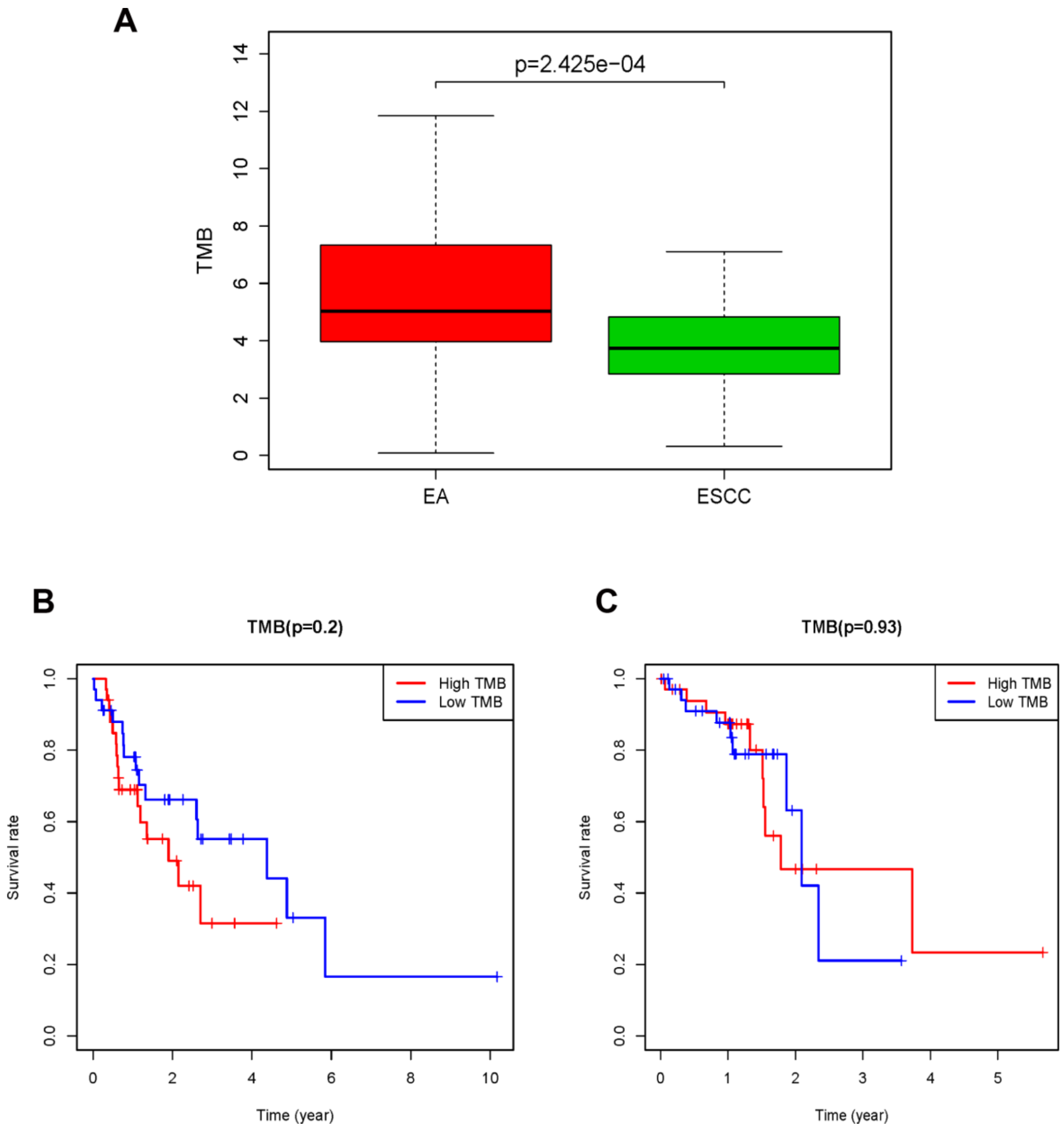

Supplementary Figure 3. The TMB level in different EC subtypes. (**A**) The level of TMB in EA was significantly higher than that in ESCC; (**B**, **C**) There was no significant difference of OS between the 2 tumor subtypes.
